# A Delphi study and development of a social and emotional wellbeing screening tool for Australian First Nations Peoples living in the Torres Strait and Northern Peninsula Area of Australia

**DOI:** 10.1371/journal.pone.0306316

**Published:** 2024-06-27

**Authors:** Kathryn Meldrum, Valda Wallace, Torres Webb, Lynne Ridgway, Rachel Quigley, Edward Strivens, Sarah G. Russell

**Affiliations:** 1 College of Medicine and Dentistry, James Cook University, Cairns, Queensland, Australia; 2 North Coast Neuropsychology, East Ballina, New South Wales, Australia; 3 Cairns and Hinterland Hospital and Health Service, Cairns, Queensland, Australia; University of South Australia, AUSTRALIA

## Abstract

Tools screening depression and anxiety developed using the Western biomedical paradigm are still used with First Nations Peoples globally, despite calls for cross-cultural adaption. Recent work by this research team found that tools used to screen for depression and anxiety were inappropriate for use with Australian First Nations Peoples living in the Torres Strait and Northern Peninsula Area of Australia. The objective of this Delphi study, the second phase of a broader four-phase project, was to gain consensus from an expert mental health and/or social and emotional wellbeing (SEWB) panel to inform the development of an appropriate screening tool. This Delphi study took place between March and May 2023. Three sequential rounds of anonymous online surveys delivered using Qualtrics^TM^ were planned, although only two were needed to reach 75% consensus. The first round sought consensus on whether a new screening tool needed to be developed or whether existing tools could be used. The second round achieved consensus. Twenty-eight experts (47% response rate) participated across the two Delphi rounds. In the second round, 83% of these experts agreed or strongly agreed that a new screening tool, using the holistic First Nations concept of social and emotional wellbeing, be developed. Ninety-four percent of them agreed that it should take a Yarning approach. These findings enabled the development of a new SEWB screening tool that adopted a Yarning (narrative) approach designed for use in primary care and geriatric settings in the region. The new tool has four different Yarning areas: Community engagement and behaviour; Stress worries; Risk; and Feeling strong. Guidelines for tool use are integrated as well as Summary and Recommendation sections. At a macro-level this project responds to the need for new screening tools that are underpinned by First Nations worldviews.

## Introduction

Australian First Nations Peoples use the term social and emotional wellbeing (SEWB) to describe and discuss their health and wellbeing. The concept of SEWB, which includes Western conceptualisations of mental health [1], is multifaceted and strengths-based [2]. There has been a significant body of work conducted with Australian First Nations Peoples to co-develop a broad conceptual model of wellbeing that encompasses quality of life, subjective well-being, and social and emotional wellbeing [3–7], as well as the role of culture in wellbeing [8]. The resulting culturally informed framework and tools provide a comprehensive assessment of the broader dimensions of wellbeing. Within this broader framework is the need for culturally appropriate screening tools to identify people with depression and anxiety who may require intervention. However, many depression and anxiety screening tools developed using the Western biomedical paradigm are still utilised with First Nations Peoples globally [9]. This is an issue because Indigenous worldviews and conceptualisation of health and wellbeing differ from the Western biomedical paradigm [10–13]. For example, the health and well-being of Australia’s First Nations Peoples is interconnected and interrelated with their communities and Country [2,5]. Additionally, concepts and words used in Western-developed screening tools may not support appropriate diagnosis and referral for First Nations Peoples [11,12].

With the aim of supporting the wellbeing of Australian First Nations Peoples, Australian research teams have been active in cross-culturally adapting [14–16], validating [17,18], and developing new tools to screen for depression [12,19] and psychological distress (depression and anxiety) [20] since the mid-2000s. This previous body of work highlights different ways that Australian First Nations Peoples’ express Western conceptualisations of depression and psychological distress and provided more appropriate tools.

The context for this study emerged from previous work that determined the prevalence of dementia in the Torres Strait and Northern Peninsula Area (NPA) of Australia [21]. Russell et al., [21] used the KICA-Dep [19] and Geriatric Anxiety Inventory [22] to screen for depression and anxiety during the dementia prevalence study. However, First Nations community members and health professionals stated that these measures were unsuitable [23] particularly the questions about suicide ideation that were offensive to the Christian beliefs of some participants. The measures also used words and concepts that were unfamiliar to participants. Subsequently a four-phase project began, aiming to develop more appropriate tools. The four phases include:

1. Conduct Yarning circles with community members and health professionals living in the Torres Strait and NPA;
2. Use a Delphi study to seek consensus on how to approach the development of a new screening tool(s);
3. Develop and pilot the new screening tool(s);
4. Validate the new screening tool(s).

Phase one used Yarning, an Australian First Nations People’s relational methodology and method [24,25]. This phase focussed on knowledge sharing about what words are used and how feelings of strong and low SEWB are described by First Nations Peoples living in the Torres Strait and NPA. Although originally aimed at developing a depression and anxiety tool, community feedback indicated that the tool be considered a SEWB tool, even though it was still focusing on these domains, whilst acknowledging that other SEWB tools developed take a broader perspective. This highlights differences in how Torres Strait Peoples and Aboriginal Peoples view the broader perspective of SEWB as it applies to their communities. Phase one has already been completed in accordance with the published protocol [26] and a manuscript discussing the findings is under review.

## Materials and methods

This paper describes phase two, a Delphi study and the subsequent development of the new SEWB screening tool. The protocol for this Delphi study has been published [27] and as described, the research team that conducted this study included an experienced Aboriginal researcher (VW) and early career Torres Strait Islander researcher (TW). The study came under the auspices of the research team’s Knowledge Circle (Indigenous Reference Group) who had oversight in all aspects of the study from conceptualisation, design and development. The aim of the Delphi study was to seek the opinion of a range of Australian First Nations mental health and/or SEWB experts and non-Indigenous clinicians with experience working with First Nations communities in this field about what approach to take to develop an appropriate screening tool for First Nations Peoples living in the Torres Strait and NPA regions of Australia. The research question that guided the first round of the Delphi was: Given the findings of the Yarning circles, should new tool(s) to screen for depression and anxiety be developed?

Ethics approval for this project was granted by the Far North Queensland Human Research Ethics Committee (HREC) (HREC/2021/QCH/73683-1518), James Cook University HREC (H8606) and Queensland University HREC (2022/HE000395). Delphi study participants were invited to participate via an email that included a link to the online survey rounds. At each Delphi round, participants were provided with written information as part of the pre-survey material. They could also download the associated Information letter. Participants gave informed consent to participate in the Delphi study by checking the “I consent to participate” box in the survey. Consequently, they were automatically directed into the online Delphi round. Participation in each Delphi round was anonymous. However, at the end of each online round participants were asked to provide their name if they wanted their contribution to the Delphi study acknowledged.

### Changes made after the protocol

**Fig 1:**
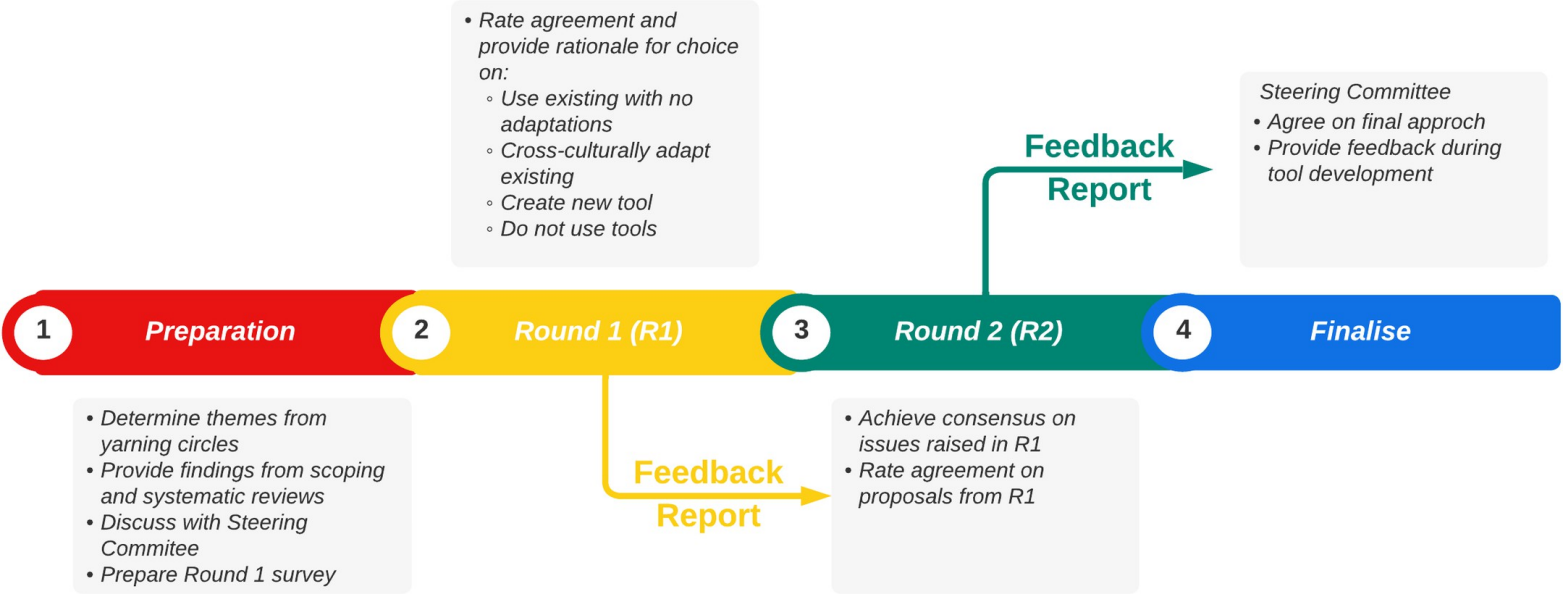
The process for this Delphi study.

While a three-round Delphi study was planned only two rounds were needed to reach consensus. The process for this Delphi is outlined in Fig 1 below.

Compilation and synthesis of background information and survey design was completed by the project lead. Subsequently, background information and surveys were piloted with other steering committee members and an independent member of the broader research team.

The Delphi study took place between March and May 2023. Each round was completed in four-week rotations. The rotation began with the survey being open online for two weeks. After the round closed, the following two weeks were used to analyse data, provide a feedback report to the steering committee, and prepare the subsequent round’s survey. To increase response rates to the second round, a reminder email was sent to participants three days before it closed.

#### Delphi round one design

To reduce completion time and minimise survey fatigue [28], both Delphi surveys were designed using the Qualtrics^TM^ survey flow function. The first survey (Delphi round one) was designed using four different response pathways. A stem statement, a conversion of the research question for the round, was used to direct participants down a pathway. The stem statement for round one was: Given the findings of the Yarning circles, new tool(s) to screen for depression and anxiety should be developed for First Nations Peoples living in the Torres Strait and NPA. Response pathways for the round one survey were:

1. Use existing mainstream screening tools.
2. Adapt previously adapted screening tools.
3. Develop a new tool.
4. Do not use screening tools.

**Fig 2:**
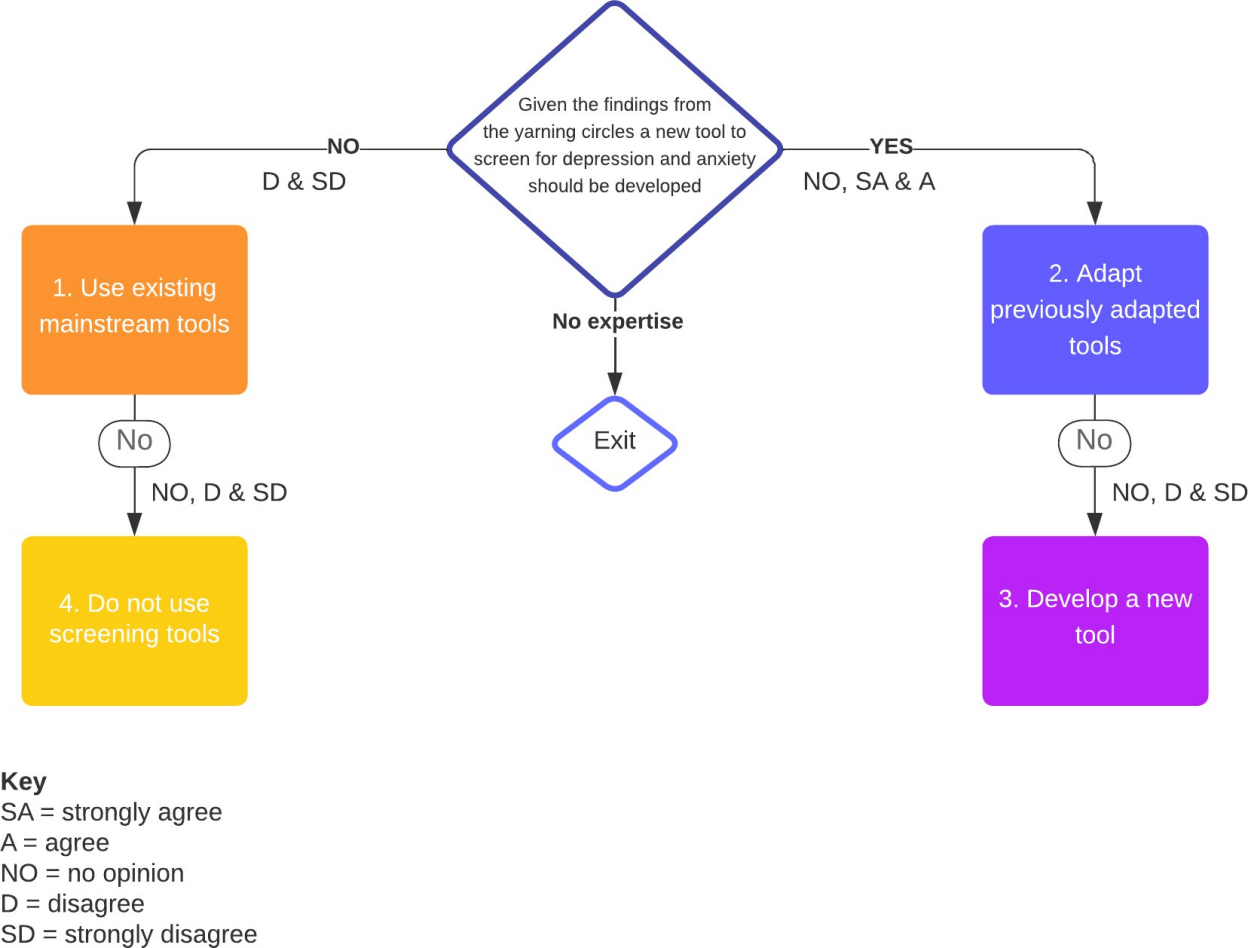
Response pathways for the round one Delphi survey.

Fig 2 illustrates how participants were directed through the survey.

Response pathways 1, 2, and 3 had examples of screening tools and associated statements related to them. At the end of the survey a broad demographic question and an article acknowledgement request were made. Finally, participants were thanked for their contribution and asked to download their responses for comparison round one’s findings. A copy of the round one Delphi survey, including the background information, is available on the Open Science Framework platform (https://osf.io/7hz6a/).

#### Data analysis

Quantitative data analysis was completed by the Qualtrics^TM^ platform. Overall results for each survey item were provided with analysis including percentages, frequency distributions, mean, standard deviation and variance in graphical and tabular forms. ‘Outside my expertise’ responses, while included in the Qualtrics^TM^ output, were not included in the subsequent report to participants or in the consensus calculation. Qualitative responses were analysed by the project lead using thematic analysis [29].

At the completion of each round the project lead compiled a report for the steering committee. The report contained the Qualtrics^TM^ output, thematic analysis of qualitative responses, a summary of the findings of the round, a report for the participants and the proposed content for the next round’s survey. The steering committee reviewed the information, piloted the next survey, and provided feedback to the project lead.

#### Delphi round two design

Following round one, the second survey (Delphi round two) was designed using two different response pathways. A stem statement, a conversion of the research question for the round, was used to direct participants down one of the tracks. The stem statement for round two was: Given the findings of round one of this Delphi study, new tool(s) to screen for depression and anxiety should be developed for First Nations Peoples living in the Torres Strait and NPA. Response pathways for the round two survey were:

1. Adapt previously adapted tools.
2. Develop a new tool.

**Fig 3:**
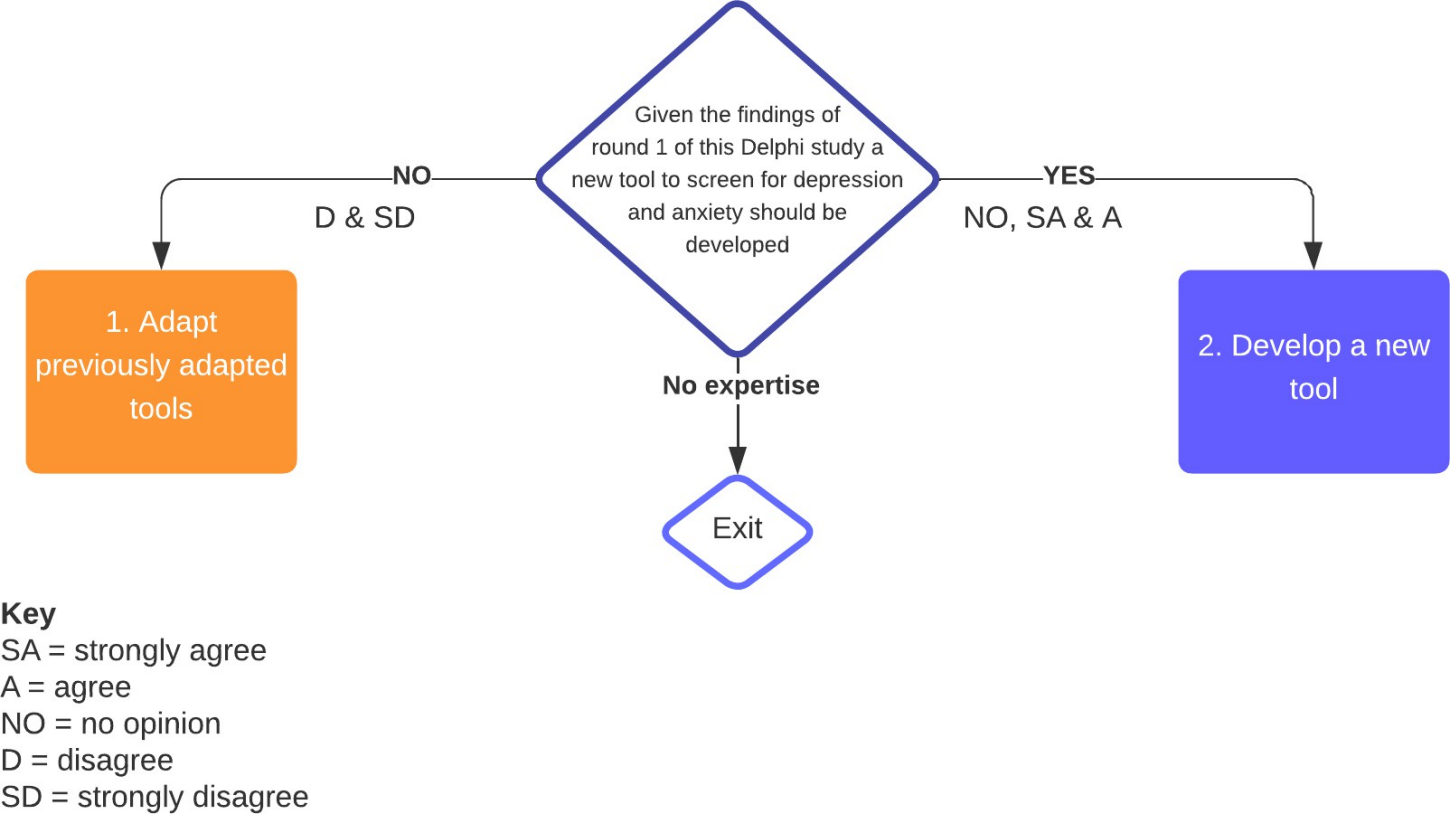
Response pathways for the round two Delphi survey.

Fig 3 outlines the response tracks for round two.

At the commencement of round two, participants were provided with a report containing results of the previous round as well as a brief infographic style summary. This information and the second survey are available on the Open Science Framework platform (https://osf.io/7hz6a/). As consensus was reached in the second round, after the data had been analysed, an infographic ‐ style summary was emailed to all the participants to provide feedback on the outcome of the Delphi study. The summary is also available for review (https://osf.io/7hz6a/).

## Results

**Fig 4:**
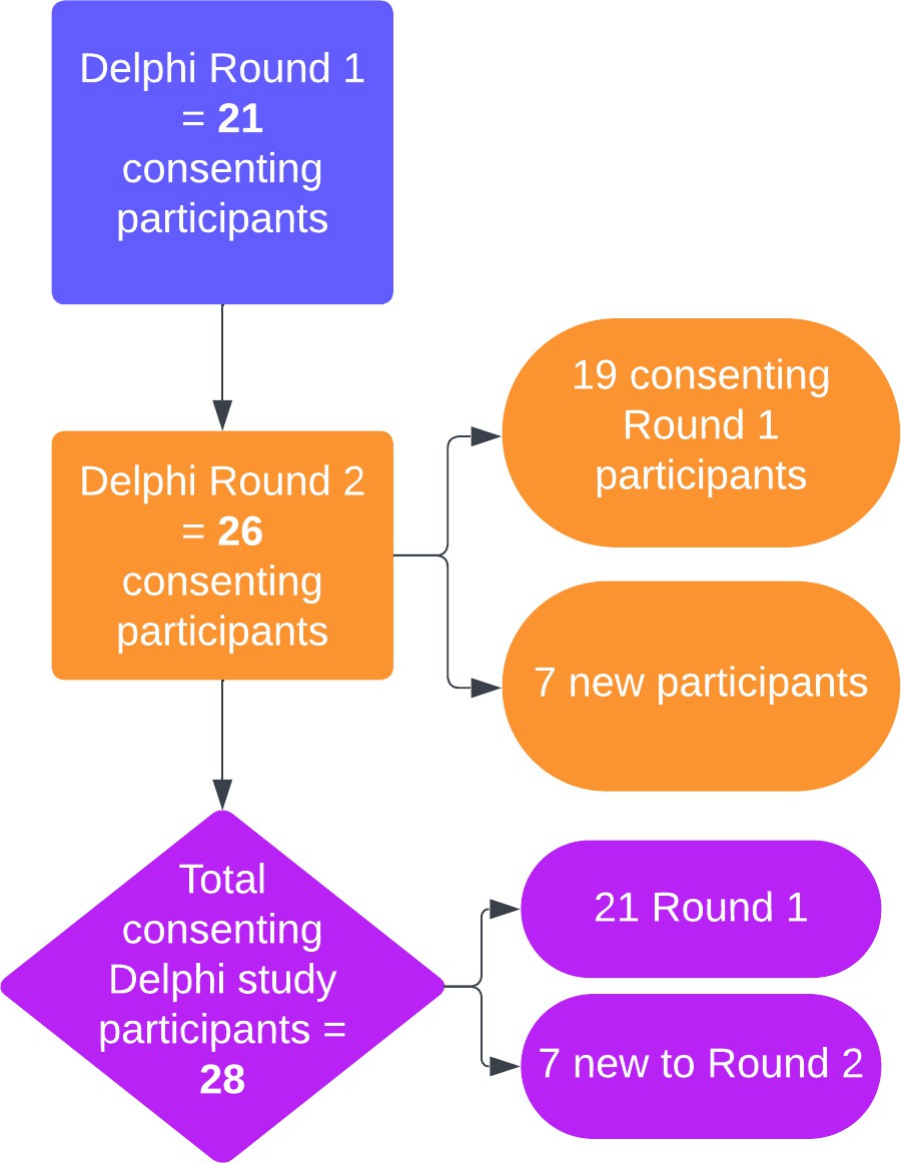
Participant numbers for rounds one and two of the Delphi study.

Overall, 28 experts (47% response rate) participated across the two Delphi rounds. Twenty-one experts (35% response rate) participated in round one, and 26 in round two (42% response rate). Fig 4 below identifies how many participants participated in each round as well as how the final participation number was calculated.

Table 1 presents the self-described role of each of the Delphi participants. Not all participants provided their ‘other’ role. However, those that did, identified themselves as geriatricians (n = 5), nurses/nurse navigators (n = 3), psychologist/researcher (n = 1) and SEWB professional (n = 1).

**Table 1:** Self-described role of Delphi study participants.

Role	Round 1 participants	Participants new to Round 2	Total	Percentage
Psychiatrist	2	0	2	7.1%
Psychologist	3	0	3	10.7%
General practitioner	2	1	3	10.7%
Mental health professional	1	0	1	3.6%
Researcher	4	1	5	17.9%
Other	9	5	14	50.0%
Total participants	21	7	28	100

### Delphi round one findings

**Fig 5:**
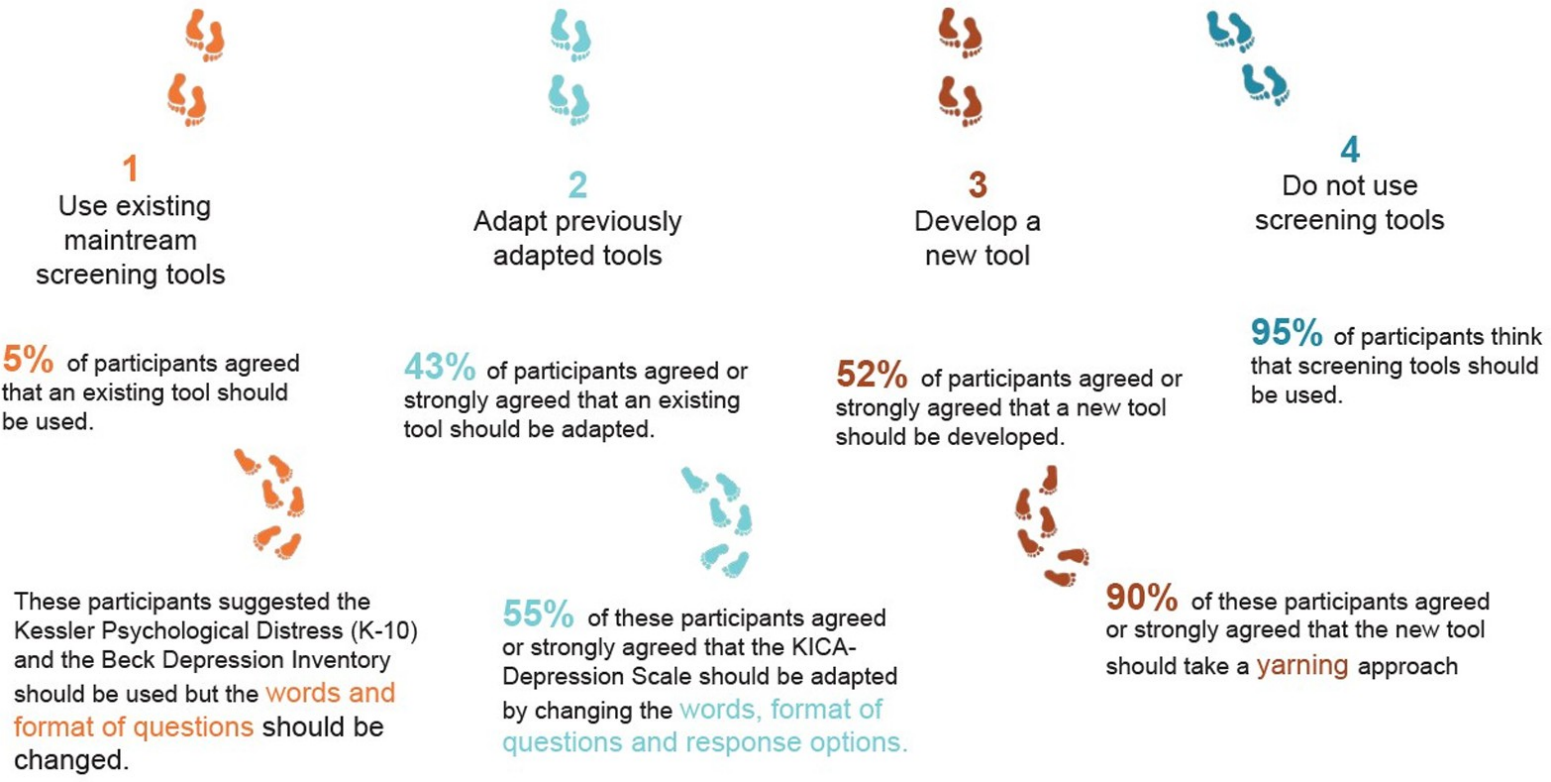
Quantitative findings for each response track in the Delphi round one survey.

Most participants opted to further adapt previously adapted tools (43%) or to develop a new screening tool (52%). Fig 5 outlines the quantitative findings from each response pathway in round one.

#### Qualitative themes for round one

Most qualitative commentary was focussed on pathways two- adapt previously adapted tools and three- develop a new tool. Themes arising from pathway two, (which included examples of the adapted PHQ-9 [15,17], KICA-Dep [19]) and adaptation of the five item Kessler Psychological Distress scale (MK-K5) [20] screening tools) included familiarity with the tool and previously reported validity with Australian First Nations Peoples.

Another theme arising from qualitative commentary was opportunities for improvements to the previously adapted tools. Participants suggested using words identified in the Yarning circles and adding a visual response scale. Examples of visual response scales included using faces or a continuum that represented feelings ranging from low–normal–high intensity or colours from green to red.

In pathway three- development of a new tool, participants were asked about what approach a new tool should take. Options included using a Yarning (narrative) approach or aa questionnaire with tick boxes option. Endorsement of the Yarning (narrative) approach confirmed it was a preferred communication style that is culturally appropriate. Yarning is adaptable and responsive to the needs of the client. For example, the length of time taken for the yarn can be flexible. Participants also commented that a Yarning approach had been suggested by participants in phase one–the Yarning circles.

Themes arising from participant’s open-ended responses (qualitative commentary) on the development of a new tool using a tick-box questionnaire style centred around the benefits and barriers. Delphi study participants identified that benefits of tick-box questionnaires included:

- Standardised approach that could prevent omissions.
- Quick to use.
- Likely to produce more consistent inter-rater reliability.
- Reduced level of expertise required to administer.

However, barriers to the use of questionnaires with Australian First Nations Peoples living in the Torres Strait and NPA included:

- Not an acceptable or appropriate approach.
- Different ways of framing SEWB so a questionnaire would need to be adapted to each region.
- Limits the information that can be obtained through open-ended dialogue.
- Issues with unfamiliar words, signs and symptoms used in other mainstream screening tools.

Another theme elicited from qualitative commentary was not limiting the new tool to either a questionnaire or Yarning approach, by developing a combined tool. For instance, by developing a prompt tool that could incorporate example questions using local words to facilitate use by non-locals. This theme was integrated into statements in round two. Finally, the general comments section at the end of the survey did not elicit additional themes.

### Delphi round two findings

**Fig 6:**
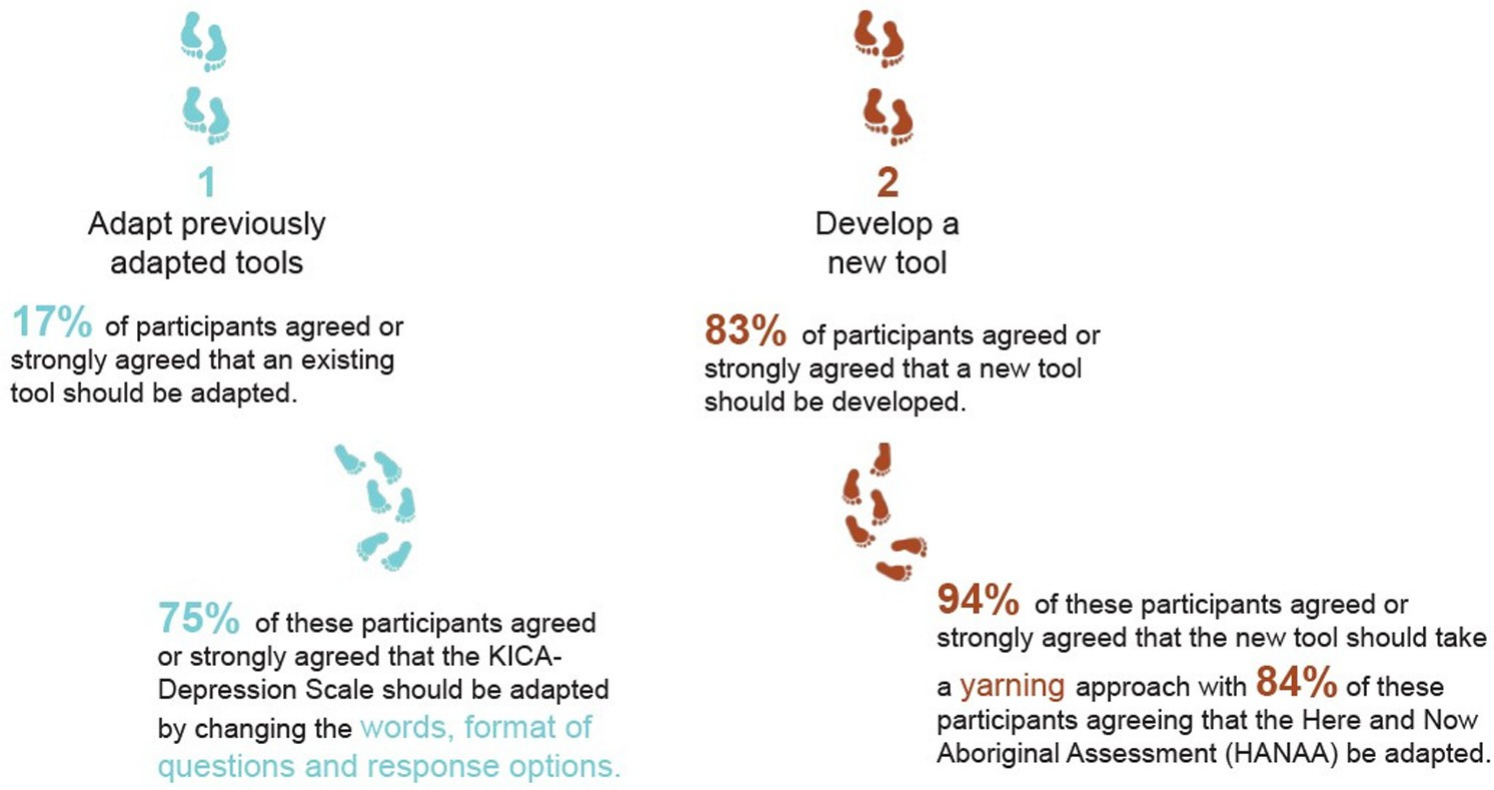
Quantitative findings for each response track in the Delphi round two survey.

Consensus was reached in round two with 83% of participants agreeing or strongly agreeing that a new tool should be developed. Fig 6 outlines the quantitative findings from each response pathway in round two.

Qualitative commentary was provided for both pathways in this round. Themes arising from pathway one focussed on which tool to adapt and suggested adaptations. Further adapting both the PHQ-9 and KICA-Dep were suggested as advantageous because they have been previously validated with Australian First Nations Peoples. Additionally, having nationally comparable tools was identified as beneficial. The suggested adaptation was to change the wording of the response scale.

In response to the theme raised in round one–to develop a combined tool, participants offered ‘for’ and ‘against’ arguments. Participants that were ‘for’ this approach indicated a combined tool provides an opportunity to:

- Capture qualitative elements that separate depression or anxiety from other mental health concerns.
- Record other ‘risk’ elements not captured in a questionnaire.

In contrast, participants ‘against’ a combination tool indicated:

- Not practical in a screening tool that should produce a numerical score/ risk assessment.
- No longer a screening tool.

Themes arising from the qualitative commentary for the second pathway, develop a new tool, were limited to the rationale for tool use and specific comments related to the Here and Now Aboriginal Assessment (HANAA) [12]. Comments on the rationale were focussed on how the tool would be used and whether screening results needed to be comparable with other cultural groups.

Specific comments on the HANAA [12] were positive, identifying that it covers the social determinants of health, and it is visually appealing and user-friendly. However, the domains were also identified as too broad for depression and anxiety. Additionally, to be used with Australian First Nations Peoples living in the Torres Strait and NPA, the HANAA [12] would need to be further adapted with appropriate words and images.

Finally, only one other theme was added from the general comments section at the end of the survey. This theme was to adapt a previously adapted questionnaire in addition to a new screening tool.

## Discussion

The aim of this Delphi study was to gain consensus from mental health and/or SEWB experts on whether a new screening tool should be developed for use with Australian First Nations Peoples living in the Torres Strait and NPA. Consensus to develop a new tool was reached in the second Delphi round with 83% of participants agreeing or strongly agreeing that this should be the approach for the next phase of this project. Ninety-four percent of these participants agreed that a new tool should take a Yarning approach and 84% of them that the HANAA [12] was suitable to adapt.

### Development of the new social and emotional wellbeing screening tool

The outcome of the Delphi study was that a new tool be developed specifically for First Nations Peoples living in the Torres Strait and NPA. The key characteristic of the new tool was that it should take a Yarning approach. Additionally, Delphi study participants indicated that the HANAA [12] was appropriate to adapt with words that were identified in the Yarning and images that were meaningful for the region. The following section describes the overarching approach for developing the new SEWB screening tool based on the results of the Yarning circles and Delphi study findings.

#### Step 1 –identify tool domains

The HANAA is a validated social and emotional wellbeing screening tool that was developed for Australian Aboriginal People living in metropolitan, regional, and remote Western Australia and the Northern Territory [12]. It is divided into a screening tool and associated guidelines. It was not our aim to replicate the HANAA [12] with specific words used by First Nations Peoples of the Torres Strait and NPA, but to use it as inspiration for developing the new SEWB screening tool.

After reviewing the findings of phase one of the broader project, the Yarning circles, four overarching themes: 1). Community engagement and behaviour, 2). Stress worries, 3). Risk, and 4). Feeling strong were identified. These themes became Yarning areas in the new tool and were subsequently compared with the ten domains of the HANAA [12] (see Table 2 below). When compared with the HANAA, the number of areas in the new tool were reduced because the Yarning circle themes and their underlying signs and symptoms ‘cut across’ several HANAA domains. For example, the physical health, sleep, mood, substance use, memory and functioning domains of the HANAA [12] were captured in the first area of the new tool: Community engagement and behaviour.

**Table 2:** Domains of the Here and Now Aboriginal Assessment [12] compared with the overarching themes from Yarning circles.

Here and Now Aboriginal Assessment		New SEWB screening tool	
Domain	Signs and symptoms	Area	Signs and symptoms
Physical health	Heart problems, kidney problems, diabetes	Community engagement and behaviour	Not active in community, have worries, sad, stress, slack, tired, waking in the night, seen no-one, staying at home, unplanned weight changes, substance use, problems thinking/doing normal activities, starting to affect normal life
Sleep	Lack of sleep, dreams, nightmares		
Mood	Worry, anger, fear, shame, stress sadness, grief, loss despair		
Substance use	Which substance/s, how much, how often, withdrawal, interpersonal problems		
Memory	Concentration, forgetfulness, difficulty remembering, confusion		
Functioning	Family, community, work, other social activities		
Suicide risk and self-harm	Thoughts, plans, attempts, cuts, burns overdoses	Risk	Feeling so low with sad or stress worries that it must end
Unusual experiences	Strange thoughts, talk or behaviour, seeing or hearing things that are not there		
Life stressors	Relationships, financial, housing, legal issues, discrimination, racism, trauma	Stress worries	Busy head, shaking, stressed out, sweaty, dizzy, breathing fast or finding it difficult to breathe, dry mouth, heart beating fast, quick to get wild
Resilience	Strengths, ways of coping, spirituality, future plans	Feeling strong	Connecting with culture through Yarning, cooking, singing, dancing, sharing food with community, going to church, praying, connecting with Island/home/Country, speaking with ancestors

**Fig 7:**
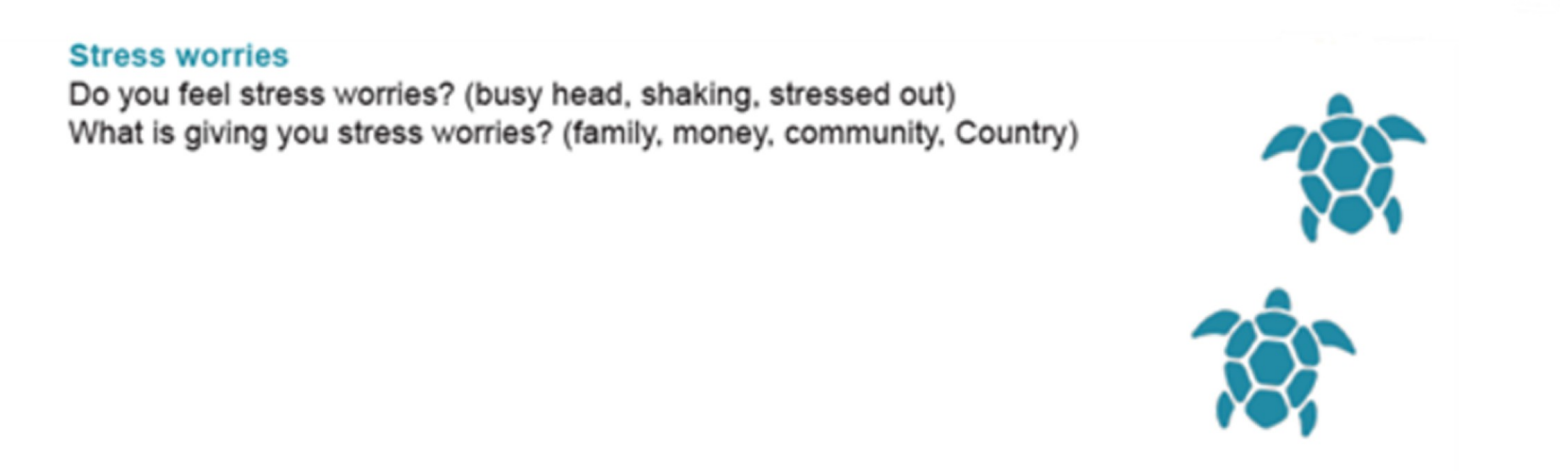
Example of the Stress worries area in the new SEWB screening tool.

Fig 7 below provides an example of the Stress worries area in the new SEWB screening tool.

To determine whether clients needed follow-up after screening, the HANAA used a dichotomous “problem/no problem” key [12]. The decision about whether a domain was a problem was made by the facilitator of the yarn. While developing the new tool we decided to take a different approach. A Summary section was included at the end of the yarn, to facilitate agreement between the client and facilitator about whether any of the areas needed follow-up. This approach was taken to ensure that the facilitator had ‘heard’ and interpreted what the client said during the yarn how it was intended. The Summary section also facilitates getting permission from the client about sharing their SEWB story with other relevant professionals as well as supporting sharing of how and when follow-up would take place.

A Recommendation section was added after the Summary so that the facilitator could note the pathways that had been agreed at the end of the yarn. Finally, Delphi study findings indicated that the image of a snake was not appropriate for the new tool. Consequently, blue coloured turtles (as illustrated in Fig 7) were added to the length of right border of the new tool.

#### Step two ‐ develop the guidelines

After the framework of the tool was developed the guidelines were written. The first version of the guidelines for the new tool followed those developed for the HANAA. Guidelines for the new tool were divided into different sections and sub-sections, including:

- Background
   ❖ How and why the new SEWB tool was developed.
- How to use the SEWB screening tool.
   ❖ Aim.
   ❖ Tool structure.
   ❖ Suggestions about setting for the yarn (outside under a tree, on the beach etc.).
- Starting the yarn
   ❖ Suggested protocol to follow to start the yarn.

After these preliminary points, the guidelines then addressed three of the four Yarning areas (Community engagement and behaviour, Stress worries, Feeling strong) by providing detail about the aim of the area and some suggested prompt questions for each. Only the aim of the Risk area was addressed in the first version of the guidelines. When the first version was subsequently circulated for Steering Committee feedback, members were asked to make detailed suggestions about what should be contained in the Risk area and how it should be written.

#### Step three–obtain feedback

**Fig 8:**
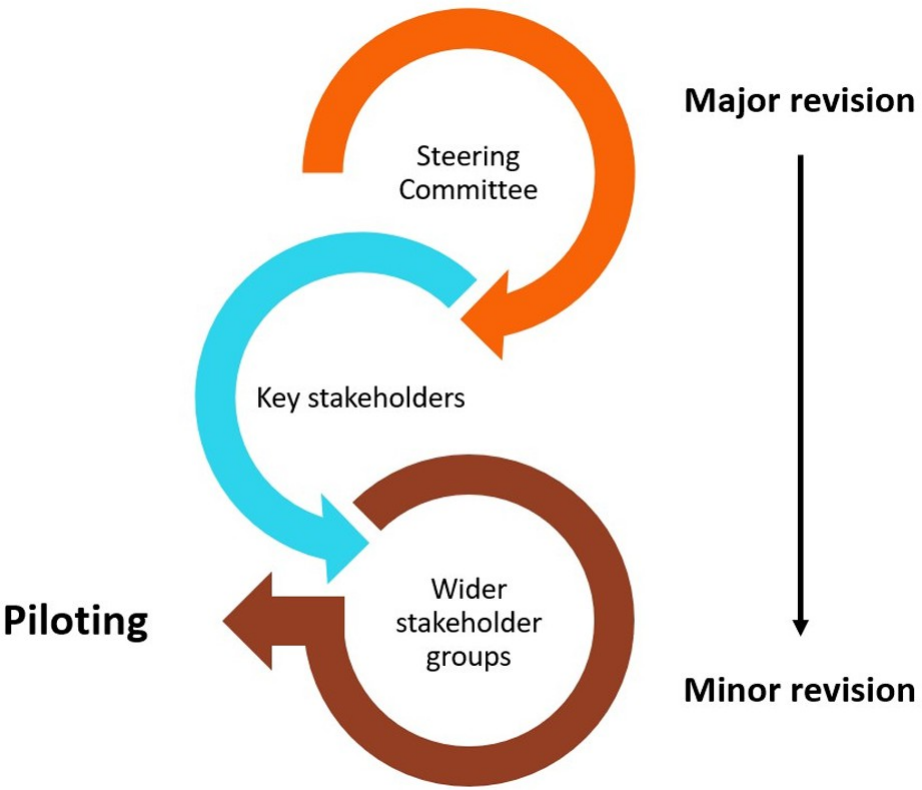
Feedback process undertaken to develop new SEWB screening tool.

The new SEWB screening tool and its guidelines were circulated in a series of feedback loops as illustrated in Fig 8 below. The first set of feedback was obtained from the steering committee who provided significant feedback on the draft screening tool and the guidelines which included the Risk area.

**Fig 9:**
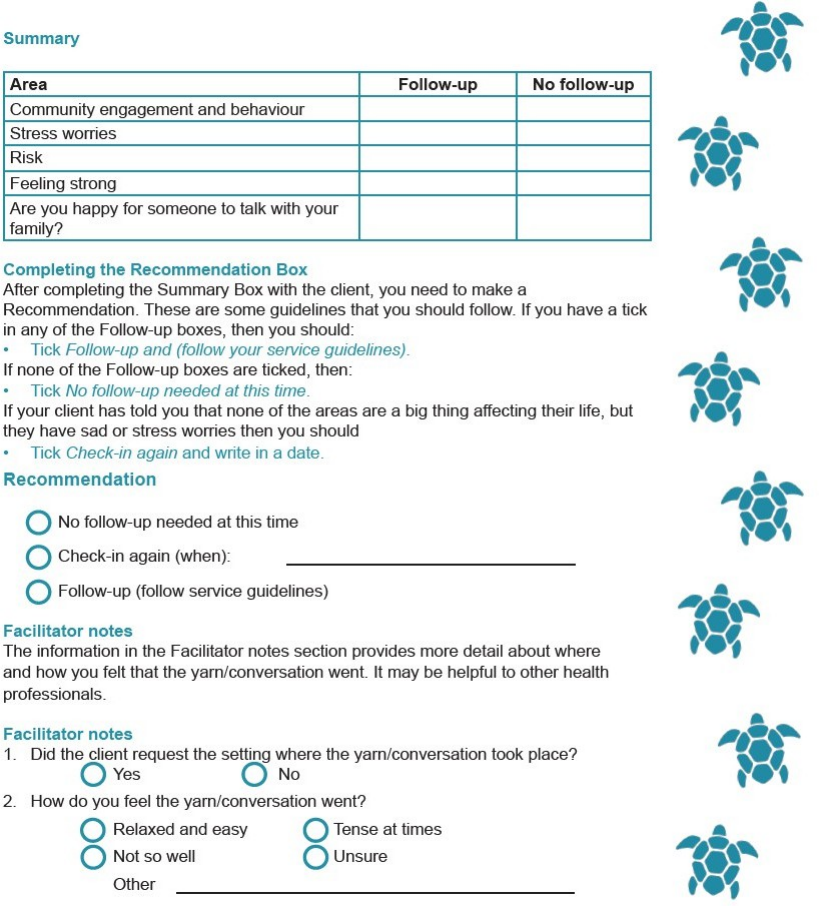
Summary and Recommendation sections of the SEWB screening tool.

Further rounds of feedback included suggestions for helping the client to feel at ease by giving them a choice about where to have the yarn and providing a drink, like a cup of tea [30], and providing a section where facilitators could note client setting requests and their feelings about how the yarn went (refer to Fig 9 below). Feedback from the steering committee was obtained for the first three versions of the screening tool and guidelines.

The second set of feedback was sought from key stakeholders which included the Mental Health and Social and Emotional Wellbeing Team for the Torres and Cape Hospital and Health Service, the HANAA authors, the research team’s Knowledge Circle and Aboriginal and Torres Strait Islander Health Workers at a small number of primary health care centres located in the Torres Strait and NPA. Key feedback integrated from these feedback rounds was:

- Integrate guidelines into screening tool so that it is one document.
- Add permission to ask family about client’s SEWB to the Summary section.
- Remove the word referral from the Summary and Recommendations sections. Just use Follow-up.

Additionally, turtles were confirmed as appropriate to use on the new SEWB screening tool as it is a unifying image for First Nations Peoples across the Torres Strait and NPA. Fig 9 below highlights some key changes to the Summary and Recommendation sections.

**Fig 10:**
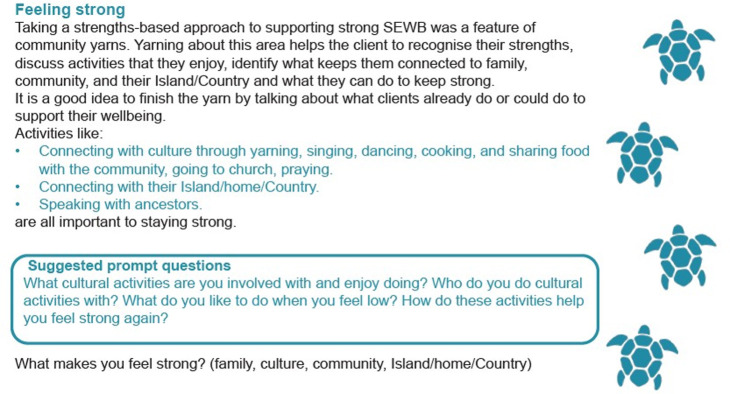
Changes made to the new SEWB screening tool after the final round of feedback.

Final feedback came from a wider stakeholder group that included Delphi study participants, staff of a private SEWB service and more Aboriginal and Torres Strait Islander Health Workers from primary health care centres located in the Torres Strait and NPA. Key feedback from these stakeholders was to reduce the amount of information in the new SEWB screening tool by summarising the key points and using colour to highlight important questions and sections. Fig 10 below illustrates how this was done for the Feeling strong area. Here, background to the inclusion of this area as well as its aim precede important information such as activities that support feeling strong in blue text. Also suggested prompt questions are highlighted in blue.

At the end of the feedback rounds the new SEWB screening tool was deemed ready for piloting.

A Delphi study is not generally used to develop screening tools [31]. However, it has been used in mental health research [32] and to develop items for a geriatric depression inventory appropriate for Chinese people [33]. A Delphi study was appropriate for this phase of the project because it sought the opinion of a broad range of clinicians, researchers and other experts working in the field of mental health and SEWB.

Häder [cited in 31] identified four Delphi study approaches: 1). The aggregation of ideas; 2). The most precise prediction of an uncertain issue; 3). Collecting expert opinions on a diverse issue; and 4). Reaching consensus. This Delphi study was novel in combining approaches 1). and 4). to answer the research question.

### Clinical implications

The outcome of the Delphi study was the development of the new SEWB screening tool for First Nations Peoples living in the Torres Strait and NPA. The new tool, designed for use in primary care and geriatric settings in the region, is now ready for piloting.

The project’s progress to date has identified that inappropriate depression and anxiety screening tools are still being used with Indigenous peoples globally [9] and that often these tools have limited validity with the populations that they were designed for [34]. Both reviews highlight practices that do not support the health and wellbeing or facilitate equitable access to diagnosis and treatment of Australian First Nations Peoples. Yarning circles also found that Australian First Nations Peoples in the Torres Strait and NPA describe and discuss SEWB differently to other groups across Australia. This Delphi study found consensus that a Yarning approach with a tool developed using SEWB, an Australian First Nations Peoples conceptualisation of health and wellbeing, is more appropriate than questionnaires developed using the Western biomedical paradigm of depression and anxiety.

### Limitations

There are two identified limitations to this study. First, that the opinion of experts who participated in the Delphi study may be different to those who were unable to participate. To mitigate the impact of this limitation, an initial invitation email was sent to experts to gauge participant numbers prior to launching the first Delphi round. If experts were unable to participate, they were asked to suggest an alternate expert. Unfortunately, this was only partially successful with low response rates. Consequently, a link to the first round was sent to all experts on the email list, apart from those who had explicitly declined the email invitation.

Second, participant drop-out between rounds was a limitation. While this Delphi achieved a relatively high overall participation rate of 47%, there was drop-out between the two rounds. A reminder email bolstered participation in the second round. However, only 70% of first round participants also participated in the second round. It is not known whether one hundred percent participation of first-round participants in the second round may have changed the outcome.

## Conclusion

The findings of this Delphi study, a consensus to develop a new screening tool for First Nations Peoples living in the Torres Strait and NPA, supported the development of a new tool, inspired, and adapted from the HANAA [12]. The new SEWB screening tool is underpinned by Australian First Nations Peoples’ conceptualisation of health and wellbeing that is broader than Western concepts of depression and anxiety and uses a Yarning approach.

## Supporting information

S1 Checklist Conducting and Reporting of Delphi Studies (CREDES) checklist (1). (DOCX)

## References

[1] Adams Y, Drew NM, Walker R. Principles of practice in mental health assessment with Aboriginal Australians. 2014 2014. In: Working together: Aboriginal and Torres Strait Islander Mental Health and Wellbeing Principles and Practice. Canberra: Department of the Prime Minister and Cabinet. 2014. p. 271–288.

[2] Gee G, Dudgeon, P. Schultz, C., Hart, A., Kelly, K. Aboriginal and Torres Strait Islander Social and Emotional Wellbeing. In: Dudgeon PM, Milroy, H., Walker, R., editors. Working Together: Aboriginal and Torres Strait Islander Mental Health and Wellbeing Principles and Practice. Canberra: Department of the Prime Minister and Cabinet.; 2014. p. 55–68.

[3] Butler TL, Anderson K, Garvey G, Cunningham J, Ratcliffe J, Tong A, et al. Aboriginal and Torres Strait islander people’s domains of wellbeing: a comprehensive literature review. Soc Science Med. 2019;233:138–57. doi: 10.1016/j.socscimed.2019.06.004 31200269

[4] Garvey G, Anderson K, Gall A, Butler TL, Cunningham J, Whop LJ, et al. What Matters 2 adults (WM2Adults): Understanding the foundations of aboriginal and Torres Strait Islander wellbeing. Int J Environ Res Public Health. 2021;18(12):6193. doi: 10.3390/ijerph18126193 34201090 PMC8226989

[5] Garvey G, Anderson K, Gall A, Butler TL, Whop LJ, Arley B, et al. The fabric of Aboriginal and Torres Strait Islander wellbeing: a conceptual model. Int J Environ Res Public Health. 2021;18(15):7745. doi: 10.3390/ijerph18157745 34360037 PMC8345714

[6] Howard K, Anderson K, Cunningham J, Cass A, Ratcliffe J, Whop LJ, et al. What Matters 2 Adults: A study protocol to develop a new preference-based wellbeing measure with Aboriginal and Torres Strait Islander adults (WM2Adults). BMC Public Health. 2020;20:1–8. 33203391 10.1186/s12889-020-09821-z PMC7672853

[7] Howard K, Garvey G, Anderson K, Dickson M, Viney R, Ratcliffe J, et al. Development of the what matters 2 adults (WM2A) wellbeing measure for Aboriginal and Torres Strait Islander adults. Soc Sci Med. 2024:116694. doi: 10.1016/j.socscimed.2024.116694 38569315

[8] Jones R, Thurber KA, Chapman J, D’Este C, Dunbar T, Wenitong M, et al. Study protocol: Our Cultures Count, the Mayi Kuwayu Study, a national longitudinal study of Aboriginal and Torres Strait Islander wellbeing. BMJ Open. 2018;8(6). doi: 10.1136/bmjopen-2018-023861 29950481 PMC6020975

[9] Meldrum K, Andersson E, Webb T, Quigley R, Strivens E, Russell S. Screening depression and anxiety in Indigenous peoples: A global scoping review. Transcult Psychiatry. 2023:13634615231187257. doi: 10.1177/13634615231187257 37490720 PMC12130600

[10] Wilson K, Richmond C. Indigenous Health and Medicine. In: Kitchin R, Thrift N, editors. International Encyclopedia of Human Geography. Oxford: Elsevier; 2009. p. 365–70.

[11] Le Grande M, Ski CF, Thompson DR, Scuffham P, Kularatna S, Jackson AC, et al. Social and emotional wellbeing assessment instruments for use with Indigenous Australians: A critical review. Soc Sci & Med. 2017;187:164–73. doi: 10.1016/j.socscimed.2017.06.046 28689090

[12] Janca A, Lyons Z, Balaratnasingam S, Parfitt D, Davison S, Laugharne J. Here and Now Aboriginal Assessment: background, development and preliminary evaluation of a culturally appropriate screening tool. Australas Psychiatry. 2015;23(3):287–92. doi: 10.1177/1039856215584514 25944764

[13] Black EB, Ranmuthugala G, Kondalsamy-Chennakesavan S, Toombs MR, Nicholson GC, Kisely S. A systematic review: identifying the prevalence rates of psychiatric disorder in Australia’s indigenous populations. Aust N Z J Psychiatry. 2015;49(5):412–29. doi: 10.1177/0004867415569802 25690747

[14] Brown A, Scales U, Beever W, Rickards B, Rowley K, O’Dea K. Exploring the expression of depression and distress in Aboriginal men in Central Australia: A qualitative study. BMC Psychiatry. 2012;12:13. 22853622 10.1186/1471-244X-12-97 PMC3441213

[15] Brown ADH, Mentha R, Rowley KG, Skinner T, Davy C, O’Dea K. Depression in Aboriginal men in central Australia: Adaptation of the Patient Health Questionnaire 9. BMC Psychiatry. 2013;13. doi: 10.1186/1471-244X-13-271 24139186 PMC3816593

[16] Esler DM, Johnston F, Thomas D. The acceptability of a depression screening tool in an urban, Aboriginal community‐controlled health service. Aust N Z J Public Health. 2007;31(3):259–63. doi: 10.1111/j.1467-842x.2007.00058.x 17679245

[17] Getting it Right Collaborative G. Getting it Right: validating a culturally specific screening tool for depression (aPHQ-9) in Aboriginal and Torres Strait Islander Australians. Med J Aust. 2019;211(1):24–30. 10.5694/mja2.50212 31256439

[18] Esler D, Johnston F, Thomas D, Davis B. The validity of a depression screening tool modified for use with Aboriginal and Torres Strait Islander people. Aust N Z J Public Health. 2008;32(4):317–21. doi: 10.1111/j.1753-6405.2008.00247.x 18782392

[19] Almeida OP, Flicker L, Fenner S, Smith K, Hyde Z, Atkinson D, et al. The Kimberley assessment of depression of older Indigenous Australians: prevalence of depressive disorders, risk factors and validation of the KICA-dep scale. PloS One. 2014;9(4):e94983. doi: 10.1371/journal.pone.0094983 24740098 PMC3989269

[20] Brinckley M-M, Calabria B, Walker J, Thurber KA, Lovett R. Reliability, validity, and clinical utility of a culturally modified Kessler scale (MK-K5) in the Aboriginal and Torres Strait Islander population. BMC public health. 2021;21(1):1–15. 34112127 10.1186/s12889-021-11138-4 PMC8194217

[21] Russell SG, Quigley R, Thompson F, Sagigi B, LoGiudice D, Smith K, et al. Prevalence of dementia in the Torres Strait. Australas J Ageing. 2021;40(2):e125–e32. doi: 10.1111/ajag.12878 33169520

[22] Pachana NA, Byrne GJ, Siddle H, Koloski N, Harley E, Arnold E. Development and validation of the Geriatric Anxiety Inventory. Internat Psychogeriatr. 2007;19(1):103–14. doi: 10.1017/S1041610206003504 16805925

[23] Russell SG, Quigley R, Thompson F, Sagigi B, Miller G, LoGiudice D, et al. Culturally appropriate assessment of depression and anxiety in older Torres Strait Islanders: limitations and recommendations. Clin Gerontol. 2022:1–13. doi: 10.1080/07317115.2022.2086090 35694996

[24] Barlo S, Boyd WE, Pelizzon A, Wilson S. Yarning as protected space: Principles and protocols. AlterNative: An International Journal of Indigenous Peoples. 2020;16(2):90–8.

[25] Bessarab D, Ng’Andu B. Yarning about yarning as a legitimate method in Indigenous research. International Journal of Critical Indigenous Studies. 2010;3(1):37–50.

[26] Meldrum K, Andersson E, Sagigi B, Webb T, Wapau C, Quigley R, et al. How Australian First Nations peoples living in the Torres Strait and Northern Peninsula Area of Australia describe and discuss social and emotional well-being: a qualitative study protocol. BMJ Open. 2022;12(12):e067052. doi: 10.1136/bmjopen-2022-067052 36600438 PMC9772669

[27] Meldrum K, Wallace V, Webb T, Ridgway L, Quigley R, Strivens E, et al. Developing an appropriate depression and anxiety screening tool for use with Australian First Nations peoples living in the Torres Strait and Northern Peninsula Area of Australia: Protocol for a Delphi study. PloS One. 2023;18(12):e0292162. doi: 10.1371/journal.pone.0292162 38060486 PMC10703283

[28] Jünger S, Payne SA, Brine J, Radbruch L, Brearley SG. Guidance on Conducting and REporting DElphi Studies (CREDES) in palliative care: Recommendations based on a methodological systematic review. Palliat Med. 2017;31(8):684–706. doi: 10.1177/0269216317690685 28190381

[29] Clarke V, Braun V, Hayfield N. Thematic analysis. In Smith J. editor. Qualitative psychology: A practical guide to research methods. London: Sage. 2015. p. 222–248.

[30] Hunter E. The Aboriginal tea ceremony: its relevance to psychiatric practice. Australas Psychiatry. 2008;16(2):130–2. doi: 10.1080/10398560701616221 17957527

[31] Niederberger M, Spranger J. Delphi technique in health sciences: a map. Front Public Health. 2020;8:457. doi: 10.3389/fpubh.2020.00457 33072683 PMC7536299

[32] Jorm AF. Using the Delphi expert consensus method in mental health research. Aust N Z J Psychiatry. 2015;49(10):887–97. doi: 10.1177/0004867415600891 26296368

[33] Xie Z, Lv X, Hu Y, Ma W, Xie H, Lin K, et al. Development and validation of the geriatric depression inventory in Chinese culture. Internat Psychogeriatr. 2015;27(9):1505–11. doi: 10.1017/S1041610215000162 25703925

[34] Meldrum K, Andersson E, Wallace V, Webb T, Quigley R, Strivens E, et al. Approaches to the development of new screening tools that assess distress in Indigenous peoples: A systematic mixed studies review. PloS One. 2023;18(9):e0291141. doi: 10.1371/journal.pone.0291141 37682832 PMC10490875

